# Experimental Study on the Frost Resistance of Basalt Fiber Reinforced Concrete

**DOI:** 10.3390/ma17184593

**Published:** 2024-09-19

**Authors:** Yihong Guo, Jianlin Gao, Jianfu Lv

**Affiliations:** College of Aerospace and Civil Engineering, Harbin Engineering University, Harbin 150001, China; gyhhrb@hrbeu.edu.cn

**Keywords:** basalt fiber reinforced concrete, frost resistance, curing conditions, freeze-thaw damage, pore structure

## Abstract

In this paper, the effect of basalt fiber (BF) on the frost resistance of concrete under different curing conditions was investigated, and its frost resistance mechanism was analyzed. Three different curing conditions (normal curing, short-term curing, and seawater curing) were adopted, and concrete with different BF volume contents was designed. Freeze-thaw (FT) tests were carried out using the rapid freezing method to test the frost resistance of basalt fiber reinforced concrete (BFRC). Additionally, the mass loss rate (MLR), relative dynamic modulus of elasticity (RDME) change, and compressive strength reduction of specimens during the freeze-thaw cycles (FTCs) were evaluated. The results show that when the BF content is 0.15%, under normal curing, short-term curing, and seawater curing conditions, the residual compressive strength of BFRC after FTCs was increased by 5.4%, 28.1%, and 30.9%, respectively, compared to plain concrete. By incorporating BF into concrete, the development of microcracks can be effectively retarded, and damage generation during FTCs can be reduced. In addition, the microscopic morphological characteristics and pore structure characteristics of concrete further elucidate the frost resistance mechanism of BFRC from a microscopic perspective.

## 1. Introduction

Basalt fiber (BF), a type of inorganic fiber obtained from basalt, is a green fiber that does not lead to the pollution of the environment [1]. BF has higher tensile strength than aramid and polypropylene fiber [2], higher temperature resistance than glass fiber [3], and better chemical durability than steel fiber [4]. In addition, BF is low-cost and well-integrated with the matrix [5,6], which makes it a fiber-reinforced material with great potential for development.

The randomly distributed fibers in fiber-reinforced concrete can effectively improve the mechanical properties of concrete [7]. In recent years, basalt fiber reinforced concrete (BFRC) has attracted extensive attention. Compared with plain concrete, BFRC has higher tensile strength [8], higher flexural strength [9], better toughness [10], and better impact resistance in terms of mechanical properties [11], and has more excellent carbonation resistance [12], better chloride penetration resistance [13], higher sulfate resistance [14], greater alkali resistance [15], and better high-temperature resistance in terms of durability [16].

The advantages of BFRC mentioned above determine that it is more suitable for some harsh environments, such as marine and cold regions [17], in which concrete is constantly exposed to the threat of freeze-thaw cycles (FTCs) [18] and suffers from severe freeze-thaw (FT) damage. FT damage decreases mass, elastic modulus, strength, fracture toughness, and impermeability of concrete, potentially threatening the bearing capacity and safety of structure [19,20,21,22,23,24]. For these reasons, the frost resistance of concrete is closely related to the service life of structures [25], and it is more meaningful to research the frost resistance of BFRC.

Some scholars have performed research related to the frost resistance of BFRC. Jin et al. [26,27] carried out experimental research on the frost resistance of BFRC with the volume content change of BF in a non-corrosive environment. He indicated that the decreasing trend of the relative elastic modulus of BFRC tended to level off with increasing BF dosage, meaning the addition of BF improved the frost resistance of concrete. Coming to similar results, Fan et al. [28] concluded that the dynamic elastic modulus of BFRC was 1.47 times as much as that of plain concrete after 100 FTCs. Yan et al. [29] and Liu et al. [30] demonstrated that the FT damage of BFRC decreased and then increased as BF content increased. The difference is that Yan et al. showed the least FT damage when the BF content was 0.5 vol%, while Liu et al. found the least FT damage when the BF content was 0.3 vol%. Concerning the mechanical properties, Li et al. [31], Sahin et al. [32], and Kasim et al. [33] reported that the compressive strength and the flexural strength of BFRC rose with the increase of BF content under the same FTCs. Gao et al. [34,35] investigated the static and dynamic behaviors of basalt-fiber-reinforced cement-soil and basalt-fiber-reinforced cemented clay after FTCs by the static splitting-tensile test and split Hopkinson pressure bar test which showed that BF significantly increased the static and dynamic tensile strength of the mixture after FTCs. Zhao et al. [36,37] operated the digital image correlation (DIC) method to study the mechanical properties of BFRC after FTCs. They concluded that BF improved the impact resistance of concrete and inhibited the bending damage of concrete.

However, the above studies on the frost resistance of BFRC have focused on concrete under normal curing conditions. The research results lack an in-depth analysis of the complex changes of BFRC during FTCs under different curing conditions. The increasing use of BFRC in civil engineering requires detailed research on its frost resistance performance to provide a reference for the durability design, especially under harsh conditions such as insufficient hydration of concrete at an early age and seawater immersion.

This paper aims to evaluate the effect of BF on the frost resistance of BFRC under different curing conditions and the FTC environment, and to explore its mechanism through macroscopic and microscopic tests. Three series of specimens (the corresponding curing conditions were normal curing, short-term curing, and seawater curing) were fabricated. The BF content of the normal cured specimens was 0.00, 0.15, 0.30, 0.45, and 0.60% by volume fractions of concrete. The remaining two series of specimens had a BF content of 0.00, 0.15, and 0.30%. The freeze-thaw medium for the normal and short-term curing condition is fresh water, and the freeze-thaw medium of the seawater curing test series is seawater. Surface spalling, mass loss rate (MLR), relative dynamic modulus of elasticity (RDME) loss, and compressive strength before and after FTCs were tested and analyzed. The microstructure of BFRC was studied in detail by scanning electron microscope (SEM) and mercury intrusion porosimeter (MIP), and fiber bonding properties, fiber-matrix interface transition zone characteristics, pore size distribution, and porosity were tested and discussed.

## 2. Raw Materials and Experiments Methods

### 2.1. Raw Materials

**Figure 1:**
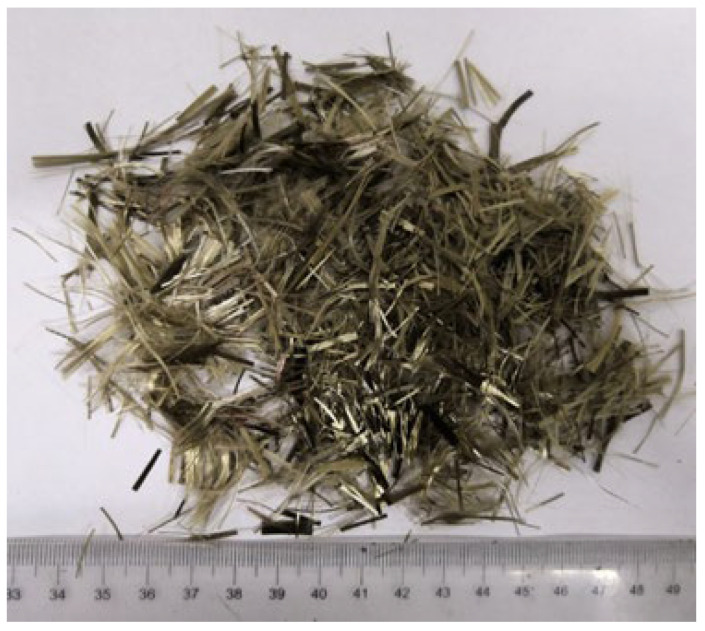
BF with a length of 12 mm.

Ordinary Portland cement (P·O 42.5) produced by YaTai Company of Harbin in China was applied for this study. Table 1 lists the chemical composition and primary physical properties of cement. Continuously graded gravel ranging from 5 to 20 mm in size was served for coarse aggregate, and river sand with a fineness modulus of 2.7 was served for fine aggregate. The polycarboxylate superplasticizer (SP) was applied to control the concrete slump of 140 mm ± 20 mm. BF produced by Hangzhou Fiber Factory of Zhejiang was used in the test (Hangzhou, China), as shown in Figure 1, and the physical and mechanical properties are given in Table 2.

### 2.2. Concrete Mix Design and Curing Conditions

The water-cement ratio of all mixtures was kept constant at 0.32. According to the different curing conditions, the specimens were divided into three series. The details of mixtures and curing conditions are presented in Table 3. Specimens of Series A and B were cured for 28 days and 7 days in a standard curing room (20 ± 1 °C and ≥95% RH) after demolding, respectively. The specimens of Series C were cured in artificial seawater at 20 ± 1 °C for 28 days after demolding. The artificial seawater was prepared concerning the actual salinity of ocean water, and the concentration was determined to be 3.5%. During this period, the seawater was changed every 14 days to ensure sufficient seawater concentration. According to JGJ/T 221-2010 [39] and JGJ55-2011 [40], five different BF contents were applied, including 0.00, 0.15, 0.30, 0.45, and 0.60% by volume fractions of concrete in Series A. The BF content of Series B and Series C were chosen as 0.00, 0.15, and 0.30% by volume fractions of concrete based on the test findings of Series A. The amount of SP was changed with different BF volume contents to maintain the required slump values of the mixtures.

### 2.3. Testing

**Figure 2:**
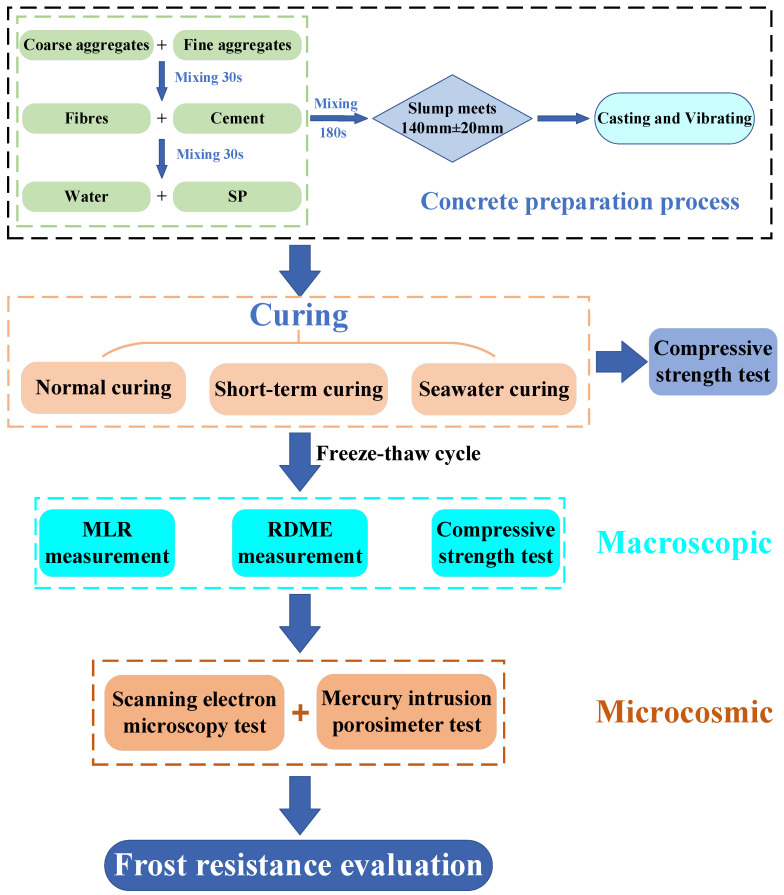
Diagram of the experimental program.

The diagram of the experimental program is shown in Figure 2. For each mix, three prismatic specimens at 100 × 100 × 400 mm were used for the FTC test, three cubic specimens at 100 × 100 × 100 mm were used for the compressive strength test after the curing was completed, and three cubic specimens at 100 × 100 × 100 mm were used for the compressive strength test after the FTC test. In order to avoid the uneven distribution of BF, the concrete preparation process in Figure 2 was strictly implemented. After casting, the mold filled with fresh concrete was placed on the shaking table and vibrated until no air bubbles appeared. All the specimens were protected from water leakage by covering them with plastic sheets until demolding 24 h later, and then the specimens were cured under the corresponding curing conditions.

#### 2.3.1. Compressive Strength Test

The compressive strength of all the mixtures was tested before and after FTCs. Each group had three specimens for testing, and the final compressive strength was taken as the average of the three test results. The experimental results were multiplied by a discount factor of 0.95 because of the size effect. The YAD-3000 test device produced by Changchun Kexin Company was adopted (Changchun, China). In order to ensure the smoothness of the specimen’s surface, the surface of the specimen was leveled with high-strength plaster after FTCs for the compressive strength test. Because of the difference in curing conditions and the decrease in compressive strength after FTCs, the machine loading rates were different. According to Chinese code GB/T50081-2019 [41], the loading rates of the machine under different conditions are shown in Table 4.

#### 2.3.2. Freeze-Thaw Cycle Test

According to the rapid frost method stipulated in GB/T 50082-2009 [42], the FTC test was carried out to study the FT damage of BFRC. CDR-3 automatic rapid freeze-thaw test equipment was used, and the central temperature of the specimens was controlled to range from −16 to 3 °C, with each cycle lasting about 4 h. In the test, the dynamic elasticity modulus of each specimen was obtained by measuring its transverse fundamental frequency using the DT-10W dynamic elasticity modulus testing.

The test was performed as follows: (i) The specimens were moved from the curing room and saturated in the corresponding FT medium. (ii) The water on the surface of the specimens was wiped clean to measure their initial mass and dynamic modulus of elasticity. (iii) The specimens were put into the specimen box in the FT chamber, and the corresponding FT medium was added to each specimen box. (iv) After every planned FTC, the dynamic elasticity modulus and mass were measured and recorded. (v) The mass loss rate (MLR) and the relative dynamic modulus of elasticity (RDME) were calculated with Equations (1) and (2), respectively, according to GB/T50082-2009. The FTC test continued until the planned number of cycles was reached, until its RDME was less than 60%, or until its MLR was greater than 5%. If the surface of the specimens was severely damaged, then the dynamic modulus of elasticity was influenced by the change of specimen mass. Then, the RDME was calculated using Equations (3) and (4).

(1) $$\Delta W_{ni}=\frac{W_{0i}-W_{ni}}{W_{0i}}\times 100$$

where Δ*W_ni_* is the MLR of the *i*th specimen after *n* FTCs (%), *W*_0*i*_ is the mass of the *i*th specimen at 0 FTC (g), *W_ni_* is the mass of the *i*th specimen after *n* FTCs (g).

(2) $$P_{i}=\frac{f_{ni}^{2}}{f_{0i}^{2}}\times 100$$

where *P_i_* is the RDME of the *i*th specimen after *n* FTCs (%), *f_ni_* is the transverse fundamental frequency of the *i*th specimen after *n* FTCs (Hz), and *f_0i_* is the initial transverse fundamental frequency of the *i*th specimen at 0 FTC (Hz).

(3) $$E_{di}=13.244\times 10^{-4}\times W_{i}L_{i}^{3}f_{i}^{2}/a_{i}^{4}$$

(4) $$P=\frac{E_{d}}{E_{d0}}\times 100$$

where *E_di_* is the dynamic elastic modulus of the *i*th specimen during the FT test (MPa), *W_i_* is the mass of the *i*th specimen during the FT test (kg), *L_i_* is the length of the *i*th specimen during the FT test (mm), *f_i_* is the transverse fundamental frequency of the *i*th specimen during the FT test (Hz), and *a_i_* is the side length of square section of the *i*th specimen during the FT test (mm).

#### 2.3.3. SEM Test

To observe the microscopic surface of BFRC and clarify the mechanism underlying its macroscopic properties, the microstructure of BFRC was examined using SEM. SEM is a common and effective method to study the microstructural morphology of cementitious composites [43,44]. Before the SEM test, the specimens were crushed into small pieces and then were dried in a vacuum desiccator at 60 °C until a constant weight was reached. Then, the pieces were sprayed with gold by an ion-sputtering instrument.

#### 2.3.4. MIP Test

MIP is a widely employed technique for characterizing the pore size distribution of cementitious materials [45]. It is a simple and fast indirect technique that yields reproducible pore size distributions. According to pore size distributions, essential characteristic parameters such as pore size, total porosity, and critical pore size are deduced. The AutoPore IV 9500 device made by the American Micromeritics Company was used to conduct the MIP test (Norcross, GA, USA). The samples were randomly selected from the specimens for each mixture and then shattered into 3–5 mm pieces. Each piece weighed approximately 4.5 g. Before testing, the samples were dried at 60 ± 1 °C for 24 h in a vacuum desiccator.

## 3. Results and Discussion

### 3.1. Appearance Changes of Concrete

**Figure 3:**
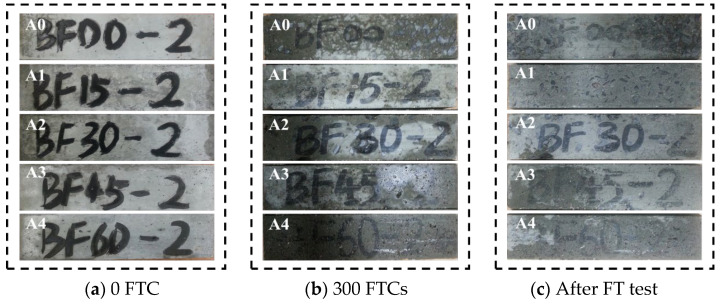
Appearances of damaged concrete under normal curing conditions.

**Figure 4:**
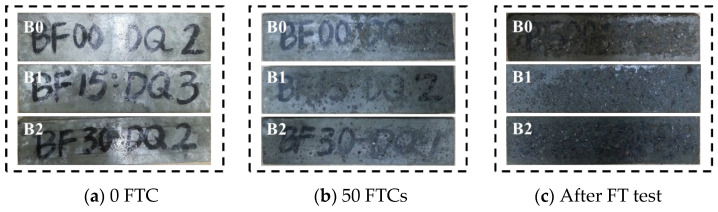
Appearances of damaged concrete under short-term curing conditions.

**Figure 5:**
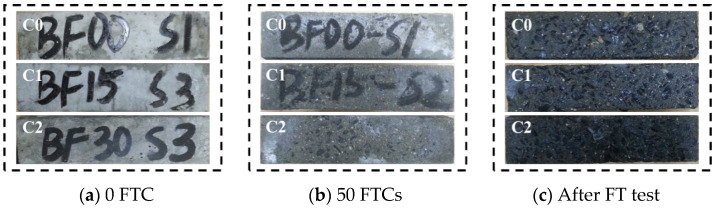
Appearances of damaged concrete under seawater curing conditions.

The FTCs were stopped according to the criteria for terminating FTCs mentioned in Section 2.3.2, and Table 5 presents the number of FTCs at the time of termination. The evaluation of concrete appearance changes is advantageous for early detection of concrete deterioration, allowing mitigation measures to be taken before the occurrence of catastrophic damage [25]. The surface spalling and pitting of each series after different FTCs are shown in Figure 3, Figure 4 and Figure 5, respectively. It can be seen from the figures that the three series of specimens showed the same process of surface spalling and pitting. Surface spalling first occurred at the edge of the concrete surface and gradually spread from the edge to the center as the FTCs rise, and finally, some of the coarse aggregates on the surface were exposed. The main reasons for this phenomenon are as follows: the edge of the concrete surface was subjected to FTCs in two or three directions at the same time; the concrete surface had a more pronounced range of temperature change than the center of concrete [46,47]; there were water-bearing pores inside concrete, and the water expanded in volume when freezing, resulting in the pore being subjected to expansion pressures [48]. 

For the appearance changes of Series A shown in Figure 3, the surface spalling and the coarse aggregate exposed in specimen A0 are obvious and severe. Compared with specimen A0, other specimens experienced less surface breakage, with only a tiny amount of mortar layer spalling and no apparent traces of exposed coarse aggregates, which proved that BF effectively improved the frost resistance of concrete. The constraining effect of BF on the matrix effectively resists the shedding of the matrix on the surface of the specimen in the FT test [49], thus slowing down the breakage of the specimen.

Compared with that of Series A, the appearance of Series B suffered more severe damage (as shown in Figure 4). The surface damage of specimens B1 and B2 was relatively uniform, spalling uniformly from the entire surface of the specimen everywhere with a shallow spalling layer, while the spalling of specimen B0 gradually broke deeper from the surface to the inside of the area with a deeper spalling layer.

The appearance changes of Series C shown in Figure 5 broke more severely and quickly than the other two series. At the termination of the test, the concrete surface showed severe spalling with serious mortar falling off, exposed aggregate, and white crystal precipitation. Hao et al. [50] observed a similar disruptive situation. After 100 FTCs in a freezing solution of 3.5% NaCl, severe spalling of the mortar occurred, and the gravel was naked. Infiltration pressure caused by the migration of supercooled water in the salt solution to the ice boundary, as well as sodium chloride crystal deposition leads to the expansion of fine cracks inside concrete, and the fine crack expands and connects to form a mesh crack structure which brings about the above-mentioned situation.

### 3.2. MLR

**Figure 6:**
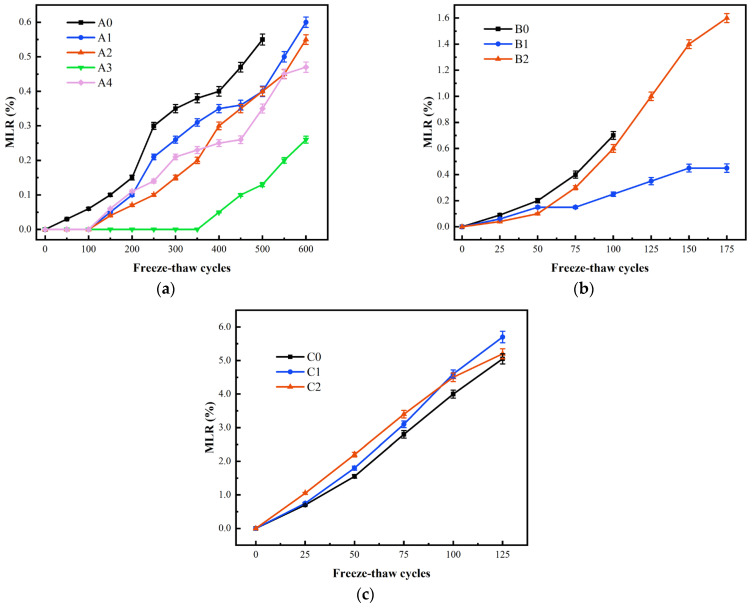
MLR results (**a**) Series A; (**b**) Series B; (**c**) Series C.

MLR is used to evaluate the degree of FT damage to specimen surface scaling [51]. The initial mass of all specimens before the FT test is presented in Table 6. The relationship between the variation of MLR and the number of FTCs under different curing conditions is given in Figure 6. The MLR gradually increased with the growth of FTCs. On the one hand, during FTCs, there is expansion pressure inside concrete leading to concrete cracking, especially in the surface layer of concrete; on the other hand, due to the small thermal conductivity of concrete, temperature stress will be generated, which leads to a gradual spalling of concrete from the surface layer to the interior. Normally, the incorporation of BF reduces the mass loss of concrete during FTCs, as shown in Figure 6a,b. BF, playing a certain inhibitory effect on mass loss, evenly scatters along the different directions in concrete, which enhances the concrete linkage ability and reduces the deterioration and shedding of surface materials. However, Figure 6c gives the opposite result about the influence of BF on MLR, which means that seawater curing has a great impact on MLR.

In Figure 6a, the MLR of A0 was the largest, reaching 0.55%, while the MLR of A3 was the least (0.13%), which was 76.4% less than that of A0. Obviously, BF enhanced the frost resistance of concrete in Series A. The sequence of MLR from small to large is A3, A4, A2, A1, and A0. BF proportion is too small to express the effect obviously, and too large so that more harmful pores and small cracks arise to reduce the compactness of concrete. In the FT environment, the denser the concrete, the less likely the external water enters concrete through its internal pores. Additionally, the smaller the hydrostatic pressure, osmotic pressure, and freezing expansion stress generated by the pore water, the less likely the specimen will be damaged [52]. Therefore, BF has both beneficial and negative effects on the frost resistance of concrete. The more connected pore is caused by high fiber dosage, but a higher fiber dosage can release pressure caused by water in different phases. The favorable effect of fibers in A3 and A4 is greater than the negative effect of high fiber dosage, resulting in less dense concrete; thus, the MLR is lower than that of the A1 and A2.

In Figure 6b, with the change in fiber content, the MLR of Series B showed a similar law to that of Series A. After 100 FTCs, the MLR of B0 was the largest, 0.7%, and the MLR of B1 was the smallest, 0.25%. Under the same number of FTCs, the MLR of Series A was smaller than that of Series B. M.J. Setzer [53] proposed that the freezing point of gel pores in concrete was lower than −43 °C due to their small diameter, and it was assumed that the water in the gel pores never froze in the tested environment. When the water in the large pore freezes, it produces volume expansion, and then the water in the capillary pore with high saturated vapor pressure will flow in the direction of the large pore with low saturated vapor pressure and continue to freeze, causing concrete to crack and become damaged. The shape of the capillary pore is diverse, interconnected, and randomly distributed. In a high hydration degree of concrete, the capillary pore is filled with hydration products, resulting in pore reefing that reduces the possibility of flow, which lowers the FT damage to concrete. The hydration reaction of Series A is sufficient, and the hydration products fill microporosity, giving concrete better frost resistance.

Compared with Series A, the MLR of Series C shown in Figure 6c was much larger. The MLR of Series C at the termination of the test was more than 5%, and the MLR of C1 and C2 were slightly higher than that of C0. Li et al. [31] stated that the MLR of BFRC under normal curing conditions was lower than that of plain concrete after chloride salt FTCs, which was different from the results of this experiment, indicating that the curing conditions had a significant effect on the frost resistance. During seawater curing, many chloride salts entered inside the concrete, allowing the concrete to reach a water-saturated state more quickly, increasing the concrete spalling significantly. With the incorporation of BF, cracks and pores existed around the fibers in the matrix [54,55], which increased the invasion of chloride salts. This negative effect is greater than the positive effect of fiber toughening, so there is a phenomenon that the amount of spalling of concrete mixed with fiber increases instead.

### 3.3. RDME

**Figure 7:**
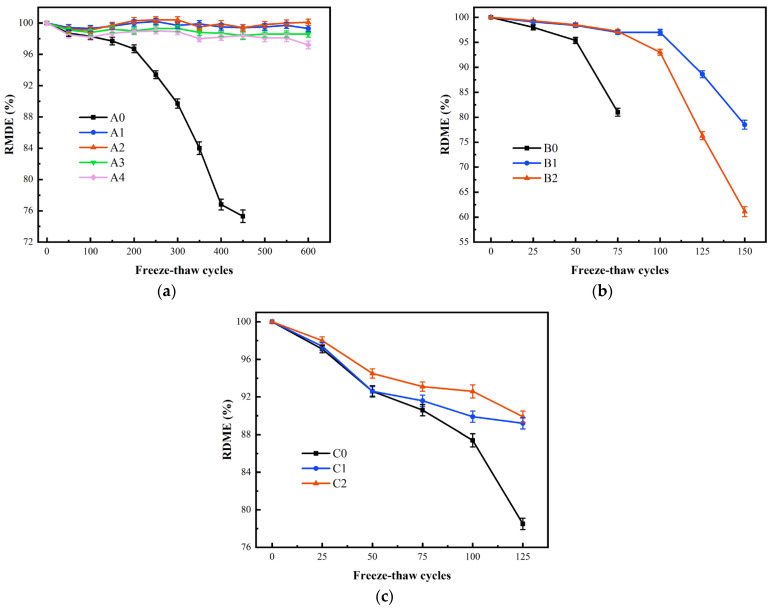
RDME results (**a**) Series A; (**b**) Series B; (**c**) Series C.

The frost heave of pore water caused by FTCs alters the internal structure of concrete, and the RDME can accurately reflect the extent of internal concrete damage. Therefore, the RDME is used to measure the damage to concrete under the action of FTCs [56]. The relationship between the variation of RDME and the number of FTC under different curing conditions are shown in Figure 7.

In Figure 7a, with the increase of FTCs, the RDME of A0 decreased continuously, and eventually by a significant amount; however, the RDME of the specimens added with BF fluctuated slightly and did not change much. In summary, the combination of BF and matrix effectively resists the adverse forces that occur during FTCs, reduces the penetration of pores and the expansion of microcracks, and improves the toughness of concrete [57]. At the same time, the continuous hydration reaction plays a particular damage-healing effect [58]. Therefore, the internal damage of BFRC is less, and the RDME loss is less and fluctuates with the increase of FTCs.

In Figure 7b, different from Series A, when the test was nearing completion, the RDME of Series B appeared to decrease abruptly, indicating that it was damaged. Because of the short curing age, the cement hydration was not sufficient. The specimens were damaged after experiencing relatively less FTCs. At this time, the adverse effect of pore water did not produce a large amount of cumulative damage. However, the fatigue stress caused by temperature alternation resulted in continuous expansion of the micro-crack. When the crack extended to a certain extent, concrete no longer withstood the fatigue stress effect and then suddenly became damaged. A suitable BF content resists a portion of fatigue stress, limits the expansion and penetration of microcracks, and increases the toughness of concrete. However, excessive BF content brings defects to concrete and increases the adverse effects of pore water. Therefore, the RDME of B1 is greater than that of B2.

In Figure 7c, C0 exhibited a significant increase in the rate of decrease of RDME before the conclusion of the test, indicating brittle damage. In contrast, C1 and C2 showed a more stable rate of decreased RDME. The higher the fiber volume rate, the greater the RDME in Series C. The artificial seawater medium has both positive and negative effects on the internal structural changes of concrete compared to the freshwater medium during the FTCs. On the one hand, the chloride salt in the solution helps concrete resist freezing by lowering the freezing point [59,60]. On the other hand, supercooled water in the solution creates greater osmotic pressure, and the salt crystallization produced during the FTCs leads to cracks in concrete [61,62]. The RDME of C0 shows a rapid decrease because the negative effect was dominant. In addition, the RDME of BFRC decreases relatively slowly due to the limiting effect of BF on the development of damage in concrete.

### 3.4. Compressive Strength

**Figure 8:**
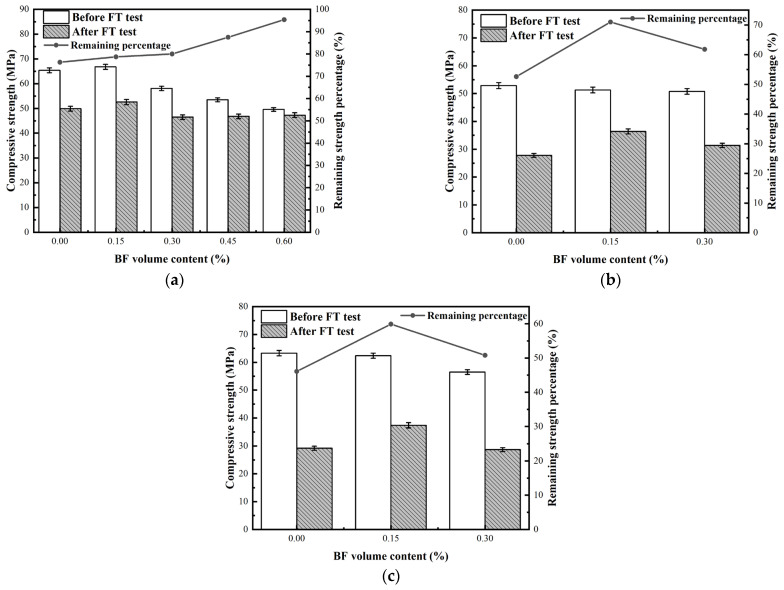
The compressive strength of specimens before and after FT test (**a**) Series A; (**b**) Series B; (**c**) Series C.

Figure 8 summarizes the compressive strength of specimens before and after the FT test and the remaining strength percentage of all specimens after the FT test. After FTCs, the compressive strength of all specimens decreased because the internal pore structure of concrete deteriorated, cracks extended, and damage became aggravated.

During the FTCs, BF restrains the matrix, slows down the rate of pore deterioration, inhibits pore expansion, and reduces the appearance of damage. Additionally, the residual strength of all the specimens was maximum at 0.15% of fiber volume under three different curing conditions.

In Figure 8a, before the FTC, the compressive strength increases and then decreases with the increase of BF volume content and reaches the maximum value at 0.15% of BF volume content. Borhan [63] and Jun and Ye [64] also discovered a similar trend in strength with increasing BF content. The addition of BF usually introduces defects in concrete, reducing concrete compactness, which has a negative impact on compressive strength, especially at higher fiber contents due to inhomogeneous dispersion. In comparison, a small amount of uniformly distributed fiber forms a mesh structure connecting the matrix, which transfers or dissipates the stress well, effectively connects the damaged patch into a whole, and stops the continued expansion of cracks [65]. When the BF volume fraction is greater than 0.15%, the positive effect is less than the negative effect, thus reducing the compressive strength.

In Figure 8b, with the increase of BF content, the compressive strength gradually decreases, and this trend is different from that of Series A. In a low hydration degree of concrete, the bond between the fiber and concrete interface is not tight enough, and the fiber plays a limited role in restraining; therefore, the early strength of BFRC decreases slightly with the increase in fiber content.

In Figure 8c, the compressive strength of Series C was slightly weaker than Series A, and the compressive strength decreased as the BF content increased before the FTC. The remaining strength percentage of Series C was the smallest of the three series after FTCs. The chlorine salt, magnesium salt, and sulfate in seawater cause the hydration products of concrete to transform into gypsum, calcium chloride oxide, Mg(OH)_2_, and other unfavorable products. Calcium chloride oxide is a very unstable compound salt, which is an important cause of concrete deterioration [66,67,68]. Chlorine salts also promote a degree of concrete satiation, making concrete FT damage more severe. As the fiber volume rate increases, more defects appear inside concrete simultaneously; the lower the degree of compactness, the more obvious the negative effects of chlorine salts, and the less the compressive strength of concrete.

### 3.5. Microscopic Structure Analysis

**Figure 9:**
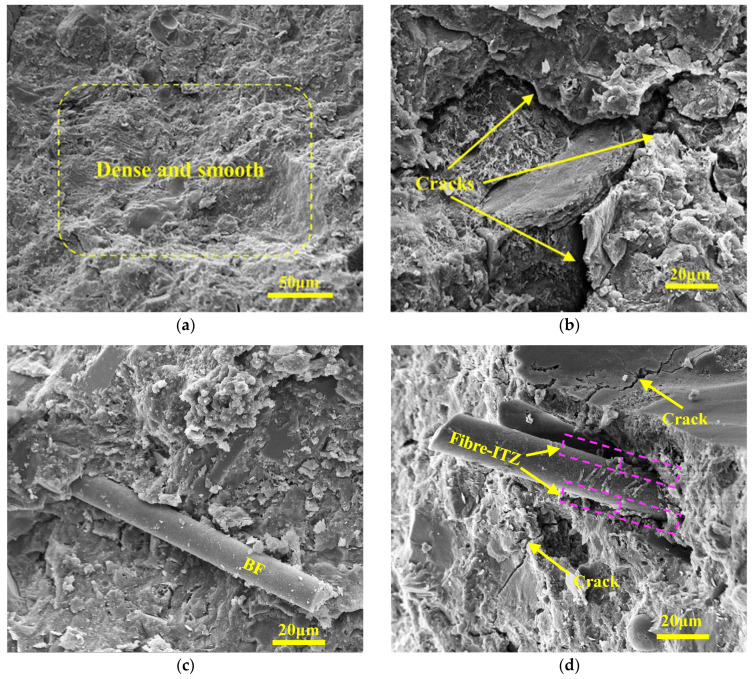
The microstructure of Series A (**a**) A0 before FTCs; (**b**) A0 after FTCs; (**c**) A1 before FTCs; (**d**) A1 after FTCs.

To analyze the enhancement mechanism of BF in concrete, crushed specimens were selected as SEM samples after the compressive strength test. Figure 9 shows the microstructure of Series A before and after FTCs. Before FTCs, the surface of the sample was flat, and the hydration products were dense. More capillary pores appeared on the surface of sample A0, but no apparent cracks appeared yet; BF in sample A1 was closely bonded with the cementitious material. BF is pulled out and ruptures in the matrix due to excessive shear friction and energy consumption [69]. After the FTCs, the hydration products on the sample surface became loose. Obvious cracks at the combination of aggregate and cementitious material appeared (Figure 9b); there was no obvious penetration cracks near the fibers and only a small number of tiny cracks (Figure 9d), indicating that reasonable fiber content plays a good role in inhibiting the extension of micro-cracks inside concrete during FTCs and enhances the frost resistance and durability of concrete.

**Figure 10:**
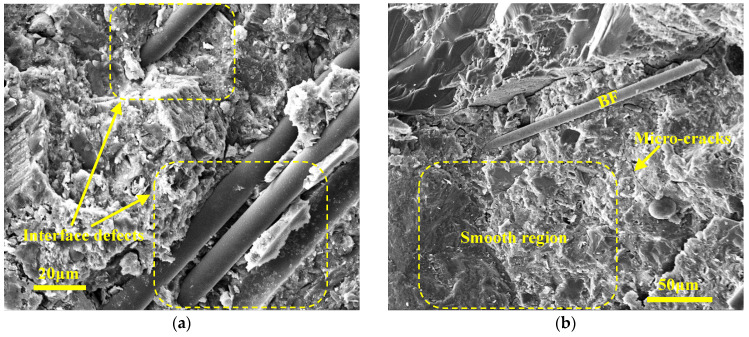
The microstructure of Series B (**a**) B1 before FTCs; (**b**) B1 after FTCs.

The microstructures of Series B before and after FTCs are given in Figure 10. Before FTCs, the sample surface was not as flat as when under normal curing conditions. The hardened cement paste has a loose structure, and the fiber’s surface is smooth. It can be inferred that curing age is not enough, which leads to small amounts of cement hydration product. After the FTCs, the microstructure of the samples looks dense because the hydration reaction was still ongoing. Meanwhile, some cracks appeared due to freezing-thawing damage.

**Figure 11:**
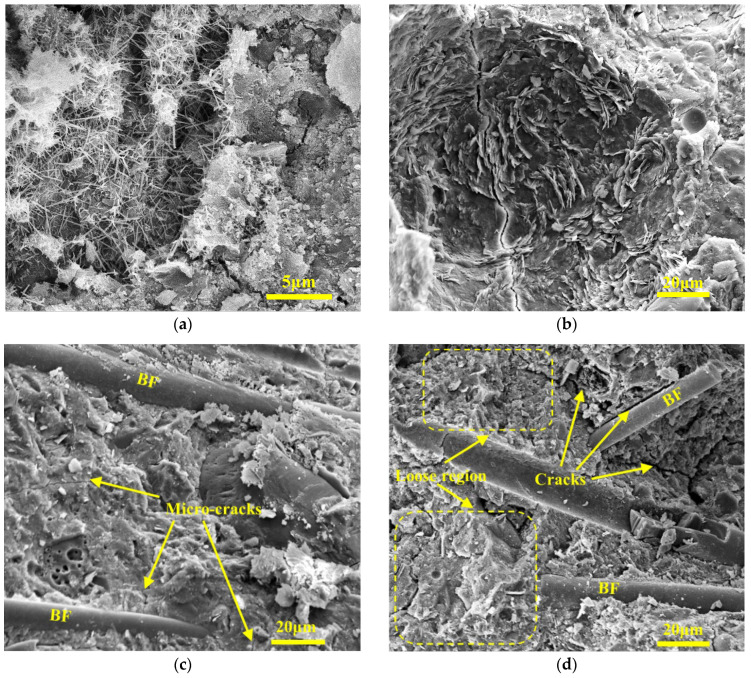
The microstructure of Series C (**a**) C0 before FTCs; (**b**) C0 after FTCs; (**c**) C1 before FTCs; (**d**) C1 after FTCs.

The microstructure of Series C before and after FTCs are shown in Figure 11. In Figure 11a, hardened cement paste has a porous structure and a needle-like crystal cover. Seawater has a high concentration of sulfate ions, which reacts with Ca(OH)_2_ to form gypsum. Gypsum further reacts to form Aft, which has a needle-like structure. As seen in Figure 11b, The surface morphology becomes a “honeycomb”. The surface is loose and porous, cracks intensify and expand, and the matrix is seriously damaged. Chlorine salts improved the degree of concrete saturation, making the concrete freeze-thaw damage more severe. With the increase in FTCs, the internal micro-cracks gradually connected to form macro-cracks, decreasing the compressive strength of concrete. Figure 11c shows that the microstructure of C1 before FTCs was dense, and the pore structure was refined compared to the normal curing samples due to the filling of the pores with Friedel’s salt, Aft, and salt crystals generated during the seawater curing process. As shown in Figure 11d, the crack extension is observed at the original location of the fibers, and the cracks are considered to be the result of stress concentration after BF debonding. In addition, the crack extension reduces the binding properties of fibers and leads to detachment between the BF and matrix [55,70,71]. BF acts as a crack restraint mechanism by using molecular adsorption force between the surfaces of cement paste and by using mechanical occlusion force between the matrix during FTCs, which hinders the extension of micro-cracks inside the matrix and stalls the occurrence and development of macro-cracks.

### 3.6. Pore Structure of BFRC

**Figure 12:**
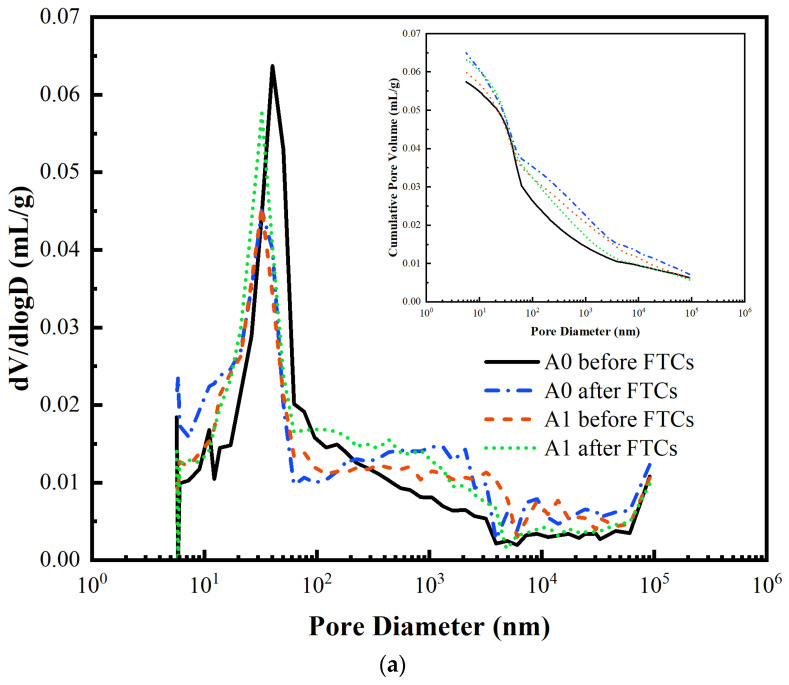

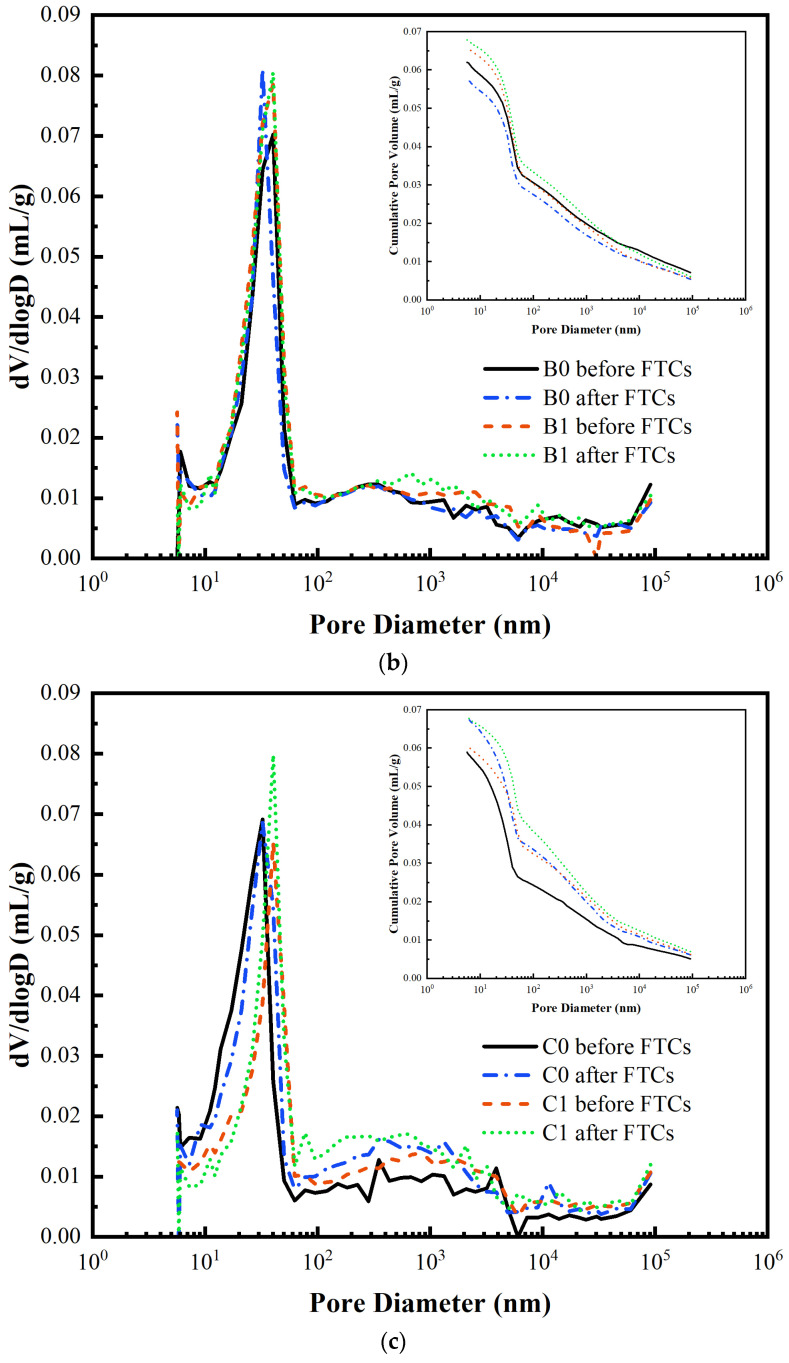
Pore size distribution (**a**) A0 and A1; (**b**) B0 and B1; (**c**) C0 and C1.

**Figure 13:**
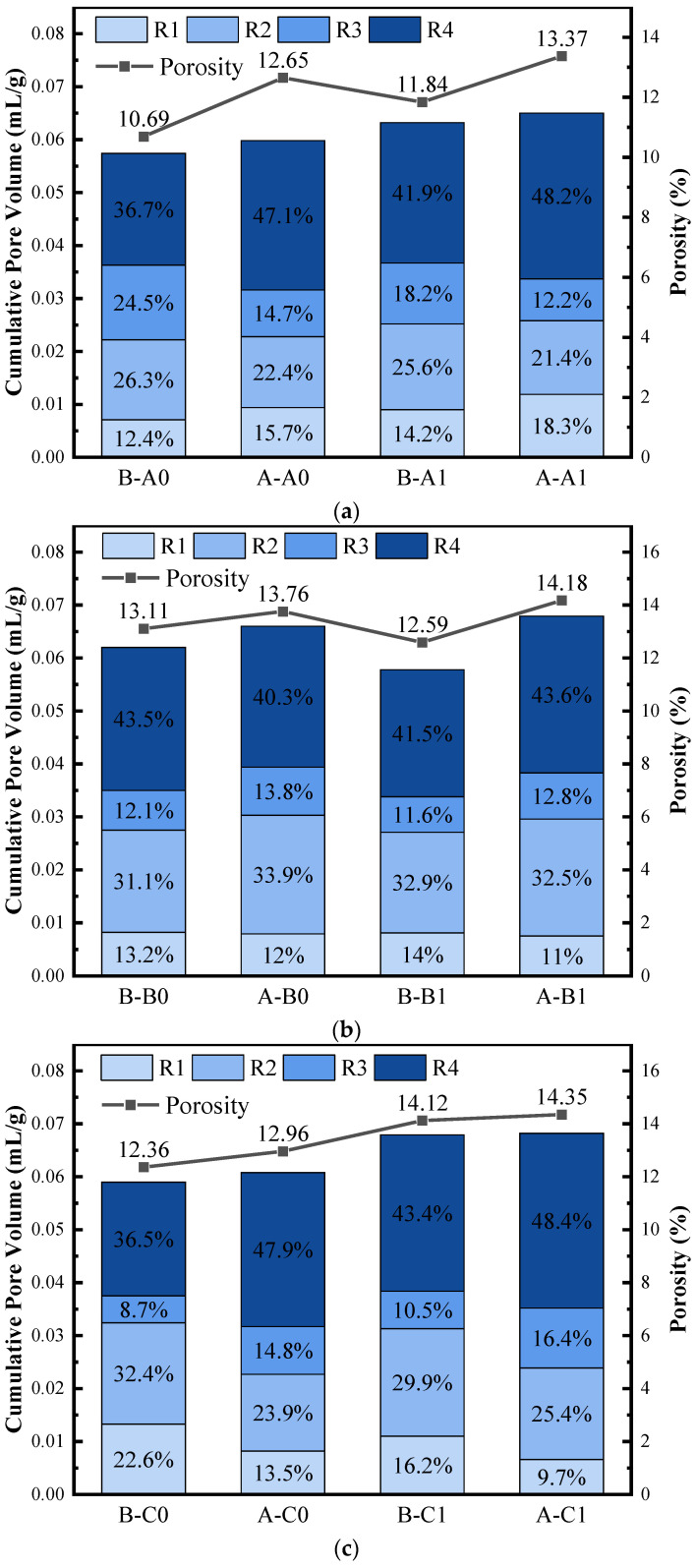
Volume percentage of pores and porosity (**a**) A0 and A1; (**b**) B0 and B1; (**c**) C0 and C1. B-A0 indicates A0 before FTCs; A-A0 indicates A0 after FTC.

To further analyze the frost resistance mechanism of BFRC, a detailed analysis of the variation of pore structure characteristics of BFRC was carried out. The variations of the pore size distribution of different series before and after FTCs are shown in Figure 12. The volume percentage of pores and porosity are displayed in Figure 13, where the pores are classified as harmless pores (R1) with diameters of less than 20 nm, minor harmful pores (R2) at 20–100 nm, harmful pores (R3) at 100–200 nm, and serious harmful pores (R4) more than 200 nm [72].

In Series A, the addition of BF alters the original pore structure. The addition of BF causes smaller pores to interconnect and become larger pores [13]. It is also possible that the fibers block some pores [49] and reduce the number of minor harmful pores. The pore size distributions of A0 and A1 have similar trends during the FTCs. The R2 and R3 pores are reduced because one part of them is transformed into the R1 pores due to the continuous effect of hydration reactions, which makes the R1 pores increase; the other part is converted into the R4 pores due to the combined effect of osmotic pressure, hydrostatic pressure, frost swelling stress, and temperature stress generated by pore water during the FTCs. Before FTCs, A1 had a greater porosity and total pore volume than A0, which corroborates with the macroscopic difference in concrete quality. After FTCs, the porosity and total mercury intake increased in A0 and A1, indicating that the pore structure was changed and the pore volume increased due to the FT damage. 

In Series B, after FTCs, B0 had reduced the R4 pores, indicating that in the FT process, the hydration reaction still continues proceeding due to short curing age, part of the R4 pores became smaller, or some of them transformed into the R2 and R3 pores. Different from B0, the R1 pores and R2 pores were decreased in B1. B1 was subjected to 75 more FTCs than B0 concrete, and the pore structure damage due to the FT action of pore water was greater in the test, so the R3 and R4 pores in concrete increased after FTCs. 

The most probable pore size of B0 and B1 did not change obviously before and after FTCs, indicating that the impermeability of concrete did not change significantly, and the adverse effect of pore water did not cause enormous FT damage during the FT test. Still, because the impact of temperature stress led to the rapid extension of cracks inside concrete, the concrete underwent brittle damage, macroscopically manifested as a sharp decrease in RDME and strength.

In Series C, after FTCs, the R3 and R4 pores of C0 and C1 specimens were increased, and the porosity and total mercury intake were increased. Compared to Series A, the FTCs in seawater resulted in a significantly higher water saturation degree of concrete than in fresh water. Although the corresponding FT time was shortened as the freezing point decreased, the freezing expansion rate was greater than that of the fresh water, and the internal pores were more significantly subjected to freeze-swelling. In addition, during the FTCs, the salt crystals inside the concrete continued to crystallize, resulting in a gradual increase in internal pore structure damage. Comparing the pore structure of C0 and C1, although the pore structure of C1 concrete is more coarse, the BF can inhibit the extension and penetration of the pores and resist some of the adverse effects of temperature stresses.

## 4. Conclusions

This paper aimed to investigate the resistance of BFRC to frost. BFRC with different BF contents were fabricated under normal, short-term, and seawater curing conditions. Freeze-thaw cycle tests, MLR, RDME, compressive tests, SEM, and MIP tests were conducted; based on the results, the following conclusions were drawn:

(i) BF can restrain the shedding of matrix material from the concrete surface during FTCs. The MLR of BFRC was significantly less than that of concrete without fiber in normal and short-term curing FT tests. In the seawater curing FT test, BFRC reached saturation more quickly and suffered severe FT damage. The fibers could play a limited role in restraining concrete, the combined effect of which led to a slight increase in the MLR of BFRC.

(ii) The limiting effect of BF on the pore penetration and microcrack extension of the matrix material can reduce the internal damage of concrete generated during FTCs. The loss of RDME of BFRC was significantly less than that of the concrete without fiber in the normal curing FT test. The RDME of BFRC decreases slower than concrete without fiber in the short-term and seawater curing FT test.

(iii) BF improves the compressive strength of concrete. Under normal curing conditions, a reasonable fiber volume rate can increase the compressive strength of concrete. In the short-term and seawater curing FT tests, the strength development of BFRC was slowed, and strength was reduced due to more defects produced by fiber incorporation, inadequate hydration, and increased chloride salt intrusion after fiber incorporation. The strength loss of concrete is suppressed due to the limitation of fiber to the concrete deterioration during FTCs.

(iv) The SEM and MIP tests analyzed the microstructural changes of BFRC during FTCs. It was found that the osmotic pressure of pore water, hydrostatic pressure, and icing expansion in the tests would cause different degrees of deterioration of the pore structure, and the temperature fatigue stress would cause the expansion and extension of microcracks. The toughening of the fibers and the limiting effect on damage development can delay the appearance of brittle damage in BFRC and improve the frost resistance of concrete.

(v) Comprehensive analysis of the change in MLR, RMDE, and compressive strength of BFRC in FTCs, under the premise that concrete has good mechanical properties, resulted in finding that the optimal fiber volume rate is 0.15% in order to improve the frost resistance of concrete.

The findings of this paper have the potential to significantly contribute towards providing theoretical support for the application and popularization of BFRC in concrete structures in high-cold regions, rapid repair of airport pavement with frost resistance requirements, and marine concrete structures. However, to ensure better frost durability of BFRC in the marine environment, further research should be conducted on improving its surface resistance to spalling during FTCs. The effect of fiber length and length-to-diameter ratio on the properties of concrete is needed. In addition, the coupling effect of basalt fibers with other fibers on the frost resistance of concrete can be further investigated. In addition, the damage model of BFRC during FTCs can be established, and the degradation mechanism of the mechanical properties and durability of BFRC after FT damage can be studied in depth.

## Figures and Tables

**Table 1:** Chemical composition and primary physical properties of cement [38].

The Chemical Composition (%)								The Physical Properties	
SiO_2_	Al_2_O_3_	Fe_2_O_3_	Na_2_O	MgO	K_2_O	CaO	MnO	Specific Gravity (kg/m^3^)	Specific Surface (m^2^/kg)
20.94	2.84	4.64	0.48	1.65	0.26	69.03	0.16	3140	350

**Table 2:** Physical and mechanical properties of BF.

Length (mm)	Diameter (μm)	Density (g/cm^3^)	Tensile Strength (MPa)	Elastic Modulus (GPa)	Interlaminar Shear Strength (MPa)	Elongation (%)	Hygroscopicity (%)
12	16	2.65	2630	88.9	56	2.99	<0.1

**Table 3:** Mixture proportions and curing conditions.

Series	Mix ID	Cement	Water	Fine Aggregate	Coarse Aggregate	BF (%)	SP (%)	Curing Conditions
		kg/m^3^						
A	A0	500	160	696	1044	0	0.92	Normal curing
	A1	500	160	696	1044	0.15	1.2	
	A2	500	160	696	1044	0.30	1.84	
	A3	500	160	696	1044	0.45	2.3	
	A4	500	160	696	1044	0.60	2.7	
B	B0	500	160	696	1044	0	0.92	Short-term curing
	B1	500	160	696	1044	0.15	1.2	
	B2	500	160	696	1044	0.30	1.84	
C	C0	500	160	696	1044	0	0.92	Seawater curing
	C1	500	160	696	1044	0.15	1.2	
	C2	500	160	696	1044	0.30	1.84	

**Table 4:** The loading rates of machine.

Series	Loading Rate (MPa/s)	
	Before FTCs	After FTCs
A	0.8	0.7
B	0.7	0.5
C	0.7	0.4

**Table 5:** The number of FTCs of specimens at the time of termination.

Specimen ID	A0	A1	A2	A3	A4	B0	B1	B2	C0	C1	C2
Number of FTCs	500	600	600	600	600	100	175	175	125	125	125

**Table 6:** The initial mass of specimens before the FT test (kg).

Specimen ID	A0	A1	A2	A3	A4	B0	B1	B2	C0	C1	C2
Initial mass	10.12	9.87	9.72	9.69	9.65	10.09	9.75	9.62	10.13	9.76	9.43

## Data Availability

Data will be made available on request.
